# Sectional Anatomy Quiz

**DOI:** 10.22038/aojnmb.2017.8707

**Published:** 2017

**Authors:** Rashid Hashmi

**Affiliations:** Rural Clinical School, University of New South Wales (UNSW), Wagga Wagga, NSW, Australia

**Keywords:** Chest, Computed tomography (CT), Sectional anatomy

## Abstract

This image based series comprises of a quiz pertaining to the identification of salient anatomical structures expected to be seen at a given level on the computed tomography (CT) followed by examples of multiple representative pathologies that can be seen at the same level in a routine clinical setting. It is expected that this will improve confidence of nuclear physicians in interpretation of the CT component of the single photon emission computed tomography (SPECT) and positron emission tomography (PET) studies.

**Figure 1:**
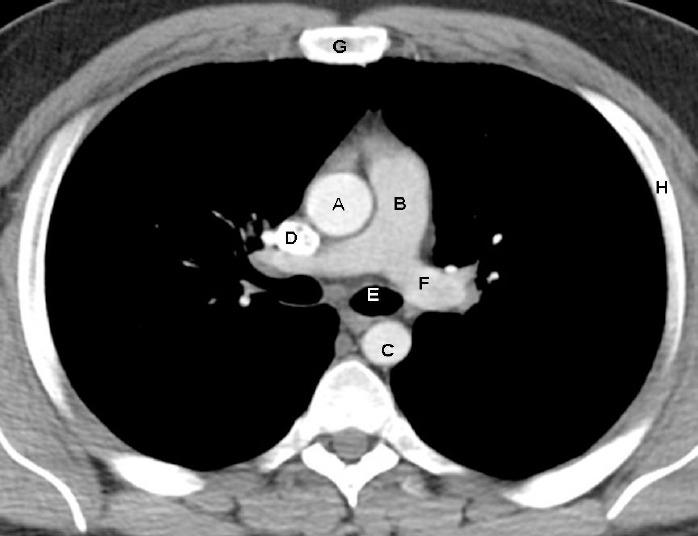
On post contrast enhanced axial CT image of the chest of a 40 years old man shown above, identify the labelled normal anatomical structures

**Figure 2:**
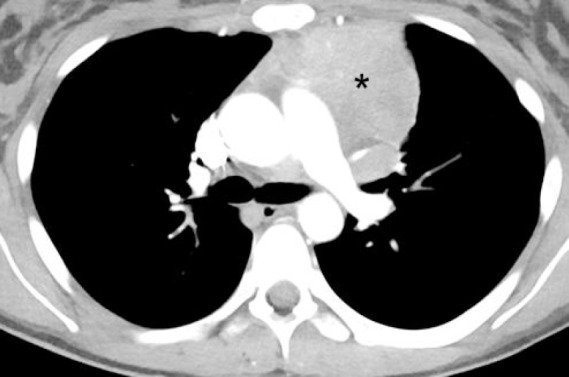
Contrast enhanced CT of the chest obtained at the level of tracheal bifurcation shows a large mediastinal mass (asterisk) anterior and to the left of the main pulmonary trunk in a patient with non-Hodgkin lymphoma

**Figure 3:**
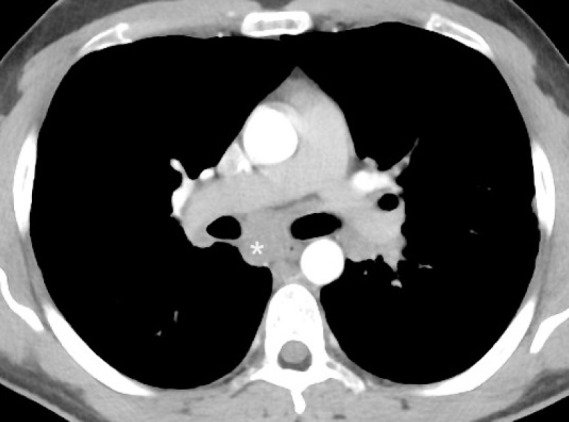
Post contrast CT of chest of a patient with history of lymphoma shows a soft tissue density mass (asterisk) in the sub-carinal region suggesting lymphadenopathy. Note that the difference in the enhancement of the aorta and pulmonary trunk and its branches is due to timing of acquisition of image after the administration of radiographic contrast

**Figure 4:**
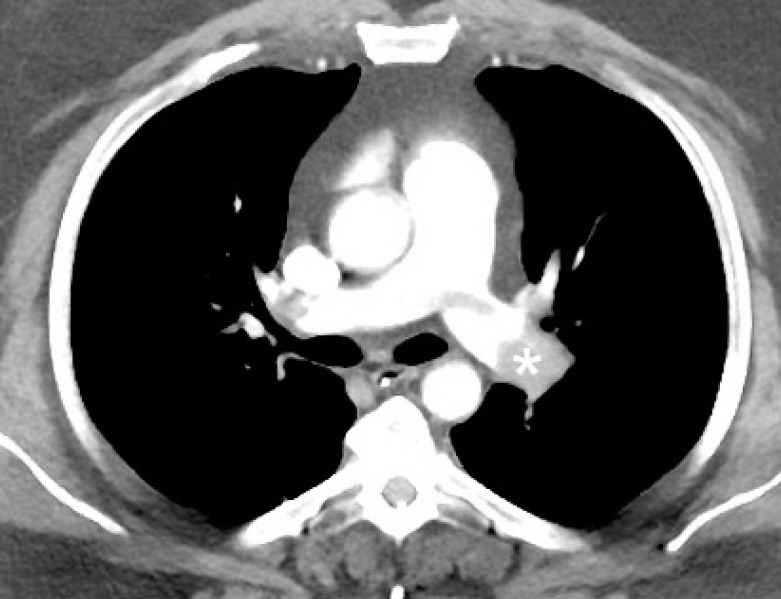
Post contrast image of CT chest of an elderly male on day 2 after a total knee replacement shows a filling defect in the left pulmonary artery (asterisk) suggesting pulmonary embolism. A small filling defect is also noted in the right pulmonary artery

**Figure 5:**
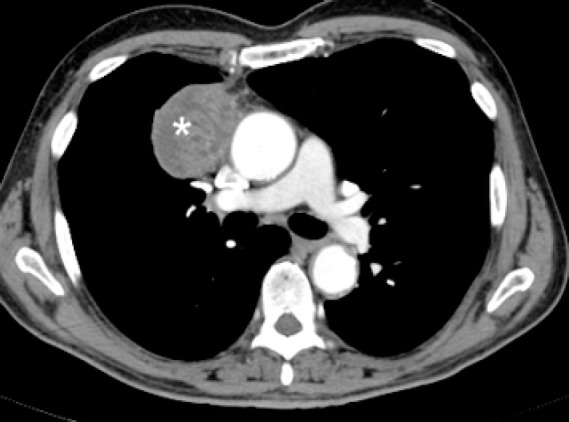
Contrast enhanced CT image of the chest of a middle aged man with an anterior mediastinal mass (asterisk) which was subsequently diagnosed to be thymoma

**POINTS TO REMEMBER**

- Tracheal bifurcation (carina) is an important landmark in sectional imaging of the chest and is approximately at the level of 4^th^/5^th^ intervertebral disk space.
- Main pulmonary trunk bifurcates into the right and left pulmonary arteries at this level with right artery larger than the left.
- Ascending and descending thoracic aorta, oesophagus and superior vena cava are the other structures that are always seen at this level.

***Answer***

The image is just below the level of tracheal bifurcation (carina) which anatomically corresponds to the level of T4/T5 intervertebral disc space posteriorly and manubriosternal junction (sternal angle or the angle of Louis).

A: Ascending aorta
B: Main pulmonary trunk
C: Descending thoracic aorta
D: Superior vena cava
E: Left main bronchus
F: Left pulmonary artery
G: Sternum
H: Rib

